# Pancreatitis of the Gastroduodenal Groove: A Case Report

**DOI:** 10.1155/2010/329587

**Published:** 2010-10-11

**Authors:** Vasileios German, Konstantinos A. Ekmektzoglou, Nicolaos Kyriakos, Paraskevas Patouras, Athanasios Kikilas

**Affiliations:** ^1^2nd Internal Medicine and Infectious Diseases Department, 401 Army General Hospital of Athens, 11525 Athens, Greece; ^2^Medical Imaging Department, 401 Army General Hospital of Athens, 11525 Athens, Greece

## Abstract

Groove pancreatitis, a form of chronic pancreatitis affecting the head of the pancreas, is localized within the groove between the pancreas head, duodenum, and common bile duct. We report a case of a male patient with groove pancreatitis who initially underwent a duodenal preserving gastrenteranastomosis. Unfortunately, the patient's symptoms were only partially controlled, necessitating a pancreaticoduodenectomy in due course as the definite surgical restoration procedure. The surgical approach selected proved inadequate since the patient's symptoms did not resolve over time. This reflects that by-pass operations like these are not indicated for the management of patients with groove pancreatitis.

## 1. Introduction

The term “groove pancreatitis”, pertaining to a form of segmental pancreatitis affecting the head of the pancreas, localized within the groove between the head of the organ, the duodenum, and the common bile duct, although coined after Stolte et al. in 1982, was first described by Becker and Bauchspeichel, almost ten years earlier [1, 2]. In 1991, Becker and Mischke classified groove pancreatitis into a pure form (involving the groove only, with preservation of the pancreatic parenchyma and the main pancreatic ducts) and a segmental form (involving both the groove and the head of the pancreas with stenosis of the pancreatic duct causing upstream dilatation) [3]. 

## 2. Case Presentation

A 34-year-old patient diagnosed with groove pancreatitis was admitted to our department because of recurrent abdominal pain, vomiting, and weight loss. The patient had been initially evaluated (6 months ago) in another hospital for similar episodes of pain and vomiting for which he had received treatment with omeprazole and pancreatic enzymes. The patient had a history of alcohol abuse for 5 years, but abstinence for the last year. Eventually groove pancreatitis was diagnosed, and duodenal stenosis by-pass surgery was performed. Unfortunately, no other information regarding imaging studies, histopathology at that time, and the selection of the aforementioned surgical procedure as the treatment of choice were available. Furthermore, the patient reported poor only symptom remission, so he sought medical advice elsewhere. 

Upon admission in our hospital, physical examination was notable for malnutrition. There was a mild decrease of bowel sounds and a moderate tenderness periumbilically. Laboratory exams showed mild leucocytosis, increased serum amylase (780 IU/L, normal range: 27–102 IU/L) and urine amylase (7940 U/L, normal range: 10–500 U/L), and mild elevation of liver function tests (Serum Glutamic Oxaloacetic Transaminase, SGOT: 113 U/L, normal range: 15–59 U/L—Serum Glutamic Pyruvate Transaminase, SGPT: 102 U/L, normal range: 10–72 U/L—Alkaline Phosphatase, ALP: 259 U/L, normal range: 38–126 U/L—Gamma-Glutamyl Transpeptidase, *γ*-GT: 118 U/L, normal range: 9–40 U/L) with normal bilirubin. Tumor marker levels (CEA, CA 19–9) were within normal limits.

**Figure 1:**
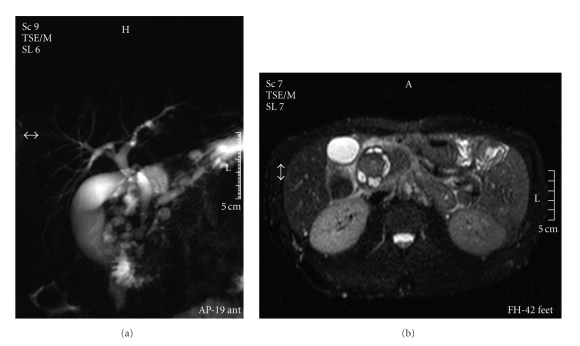
MRCP and MR images showing thickening of the second part of the duodenum, cystic formation on the duodenal wall, longitudinal narrowing of the pancreatic duct, and a mild dilatation of the common bile duct.

The patient underwent consecutively an ultrasound (US), a Computed Tomography (CT) scan, and a Magnetic Resonance Imaging (MRI) examination of the upper abdomen. A thickening of the second part of the duodenum, causing concentric obstruction of the lumen, was noted. Cystic formation on the duodenal wall was also prominent. The presence of tissue between the duodenum and the pancreas was shown. There was longitudinal narrowing of the pancreatic duct and a mild dilatation of the common bile duct. Several cystic lesions were noted on the head of the pancreas. The above findings were confirmed on the Magnetic Resonance cholangiopancreatography (MRCP) that followed (Figures 1(a) and 1(b)). 

The upper gastrointestinal endoscopy showed the gastrenteranastomosis with well functioning proximal and distal loops revealing severe edema and stenosis of the second part of the duodenum with erosive inflammation. Histological examination of the biopsy specimens from the duodenal mucosa showed mild nonspecific gastritis and hyperplastic Brunner's glands. 

The patient was discharged with his symptoms partially controlled, and pancreatoduodenectomy has been planned in due course as the definite surgical restoration procedure. He was put on a strict diet, proton pump inhibitors, and pancreatic enzyme substitutes, and he had an uneventful period of two months (he was on a stable state without experiencing any abdominal pain and with his biochemical profile—white blood cell count, serum and urine amylase, and liver function tests—returning to almost normal values).

## 3. Discussion

Although, some authors have tried to unify the concept of “groove pancreatitis”, “cystic dystrophy of heterotopic pancreas”, and “paraduodenal wall cyst” as the same clinical entity, based on distinct clinicopathological findings, under the term “paraduodenal pancreatitis”, the pathogenesis of groove pancreatitis remains controversial as several factors are implicated. While heterotopic pancreas is only occasionally found in groove pancreatitis, the presence of this feature is an inherent precondition for cystic dystrophy of the duodenal wall in the heterotopic pancreas and is characterized by the presence of cysts surrounded by inflammation and fibrosis in the duodenal wall, along with pancreatic ducts and lobules. Peptic ulcers, gastric resections, true duodenal wall cysts, and pancreatic head cysts as well as previous diseases of the biliary system are also believed to be triggering factors [1, 3–5]. Others consider the altered pancreatic secretion via the Santorini's duct and the increase of the viscosity of the pancreatic juice due to alcohol use as the major causes of the inflammation [6, 7].

Macroscopic examination of the surgical specimen usually reveals an abundant whitish firm mass of the groove area that produces a stenosis in the terminal common bile duct, with cystic lesions, either true cysts or pseudocysts, being frequently encountered in the groove or the duodenal wall. Major histopathological findings include the presence of scar tissue with fibrosis in the pancreaticoduodenal groove (pure form) or in the groove and the superior portion of the pancreatic head (segmental form). On microscopic examination, extensive fibrosis of the duodenal wall with concomitant Brunner gland hyperplasia in the submucosa can be seen. Extensive fibrosis, acinar involution, and intimal fibrosis of the pancreatic arterioles are frequently observed in the pancreatic biopsy [1, 3, 4].

The disease mainly affects middle-aged men with a preceding history of alcohol abuse. Clinical presentation resembles that of chronic pancreatitis, with postprandial abdominal pain of varying degrees. Duodenal stenosis often leads to early satiety, vomiting and weight loss. These symptoms last from weeks to months, commonly remitting upon resuming of enteric feeding. Jaundice is rare. The course of the disease is often chronic and debilitating. Blood tests often show a slight elevation of serum pancreatic enzymes and occasionally of liver function tests. Tumor markers are rarely elevated [1, 3].

Upper gastrointestinal endoscopy often reveals an inflamed and polypoid duodenal mucosa with stenosis of the duodenal lumen [8]. Endoscopic biopsy specimens, obtained from the edematous mucosa of duodenum, show marked inflammation and hyperplastic Brunner's glands. Abdominal ultrasound usually depicts a hypoechoic mass, narrowing of the second part of the duodenum, and evidence of bile duct obstruction [9]. Endoscopic ultrasound may reveal thickening and subsequent stenosis of the second portion of the duodenum on the pancreatic side along with intramural cysts. Enlargement of the pancreatic head can be described along with calcifications, pseudocysts, and dilatation of the main pancreatic duct [10]. The CT scan can reflect the histological characteristics of the disease. A hypodense, poorly enhanced mass between the pancreatic head and a thickened duodenal wall is visualized, with cysts usually seen in the duodenal wall and/or the groove as well as duodenal stenosis due to wall thickening [11]. A barium X-ray study can also provide information delineating a severe circumferential deformation and accompanying stenosis of the second portion of the duodenum [10].

MRI findings are also demonstrative of the pathologic features characteristic of this entity: the fibrous tissue in the pancreaticoduodenal groove, the duodenal wall inflammation, and the groove and/or duodenal wall cyst formation. The most characteristic finding on MRI is a sheet-like mass between the head of pancreas and the C-loop of duodenum. A T1-weighted image reveals a mass that is hypointense when compared to the pancreatic parenchyma, while a T2-weighted image reveals a hypo-, iso-, or slightly hyperintense mass. Cystic lesions are well shown in the groove or the duodenal wall, especially on T2-weighted images. Since some degree of common bile duct stenosis is almost always found, the sign of common bile duct tapering is characteristically seen in contrast to the abrupt and “shouldered” aspect of stenosis in pancreatic cancer. A progressive pattern of narrowing of the main pancreatic duct in the head of the gland can also be depicted, especially in the segmental form of the disease [12].

Endoscopic retrograde cholangiopancreatography (ERCP) or MRCP also depicts the relationship between the ductal system and the cystic changes. Duodenal evaluation is important in differentiating groove pancreatitis from pancreatic cancer, because marked inflammatory duodenal parietal thickening is not a common feature associated with tumors in the pancreatic head. ERCP can demonstrate dilatation of the Santorini's duct and its branches, depicting intraductal stones. MRCP reveals a widening of the space between the distal pancreatic and common bile ducts and duodenal lumen. Finally, while groove pancreatitis is not usually associated with a significant degree of biliary dilatation, distention of the gallbladder is usual (banana-shaped gallbladder) [12].

Regarding the differential diagnosis, in the pure form, the physician should rule out duodenal cancer, common bile duct cancer, or, even, acute pancreatitis. In the segmental form, pancreatic adenocarcinoma should be excluded [13]. Some authors favor CT, including dynamic study, as the best imaging study to demonstrate the characteristic findings of groove pancreatitis and suggest the diagnosis while others consider MRI as the best single comprehensive study to evaluate the many aspects of the disease [11, 12]. However, both the CT and MR imaging findings of groove pancreatic carcinomas can resemble those of groove pancreatitis. ERCP and endoscopic ultrasound may be used to differentiate between the above two conditions. Differential diagnosis may be achieved by the pathological diagnosis of a biopsy specimen of the duodenal mucosa, after careful consideration of the CT scan, MRCP, and endoscopic ultrasound findings suggestive of groove pancreatitis. [14].

As far as treatment is concerned, there are two therapeutic options: (1) conservative medical measures and (2) surgery. The second is the most common due to the severity of the symptoms and in order to rule out malignancy. The surgical treatment of choice is a pancreatoduodenectomy using the Whipple procedure [6]. However, other surgical procedures have been reported (pylorus-preserving pancreatoduodenectomy, pancreatojejunostomy, distal gastrectomy and Billroth II reconstruction, duodenoduodenostomy, and wedge resection) with, sometimes, fairly good results [6, 15, 16]. 

In our case, the surgical approach selected proved inadequate since the patient's symptoms did not resolve over time. This reflects that by-pass operations like these are not indicated for the management of patients with groove pancreatitis. It is our belief that such patients should be medically managed more aggressively. Radical operations, such as the Whipple procedure, should be performed whenever possible and feasible.

New imaging techniques have markedly improved the accurate diagnosis of pancreatic disease; however, it is still difficult to differentiate groove pancreatitis from pancreatic carcinoma. Groove pancreatitis presents various clinical features and, often, mimics pancreatic head carcinoma. This condition should be kept in mind in all cases of focal pancreatitis involving the region between the head of the pancreas, the duodenum, and the common bile duct. Awareness of this disease may lead to more reliable preoperative diagnosis avoiding unnecessary radical surgery. Patients with groove pancreatitis having undergone conservative treatment or pancreatic preserving operations should be carefully followed up due to the risk of coexistent carcinoma.
